# Host Species-Dependent Transmission of Tomato Leaf Curl New Delhi Virus-ES by *Bemisia tabaci*

**DOI:** 10.3390/plants11030390

**Published:** 2022-01-30

**Authors:** Dirk Janssen, Almudena Simón, Maher Boulares, Leticia Ruiz

**Affiliations:** Centro La Mojonera, IFAPA, 04745 La Mojonera, Spain; almudenasimonmartinez@gmail.com (A.S.); maher.boularesbae@gmail.com (M.B.); mleticia.ruiz@juntadeandalucia.es (L.R.)

**Keywords:** begomovirus, *Bemisia tabaci*, crop protection, zucchini, tomato

## Abstract

The tomato leaf curl New Delhi virus (ToLCNDV) is a bipartite, single-stranded begomovirus that was first identified in India in 1995 affecting solanaceous crops. A different strain, named ToLCNDV-ES, was introduced in Spain in 2012 and causes severe symptoms in zucchini crops. Virus transmission experiments with the whitefly *Bemisia tabaci*, were used to compare the transmission parameters in zucchini and tomato plants. The minimum acquisition access period and inoculation access period of ToLCNDV-ES transmission was similar in zucchini and tomato. However, the transmission efficiency was significantly higher in zucchini (96%) compared to tomato (2%). The maximum retention of the virus in the vector was 16 days. *B. tabaci* feeding on, or recently emerged from infected zucchini plants, accumulated more virus than those from infected tomato, as determined by real-time PCR. A total of 20% of *B. tabaci* that were recently emerged from infected zucchini, and none from infected tomato, were able to transmit the virus to virus-free zucchini. The results may explain the different incidences of ToLCNDV-ES in zucchini and tomato crops in Spain. But they are also relevant for ToLCNDV-ES management of crops and the role of the trade and transport of infected plant material, when small-sized immature stages of *B. tabaci* could be a source of infection.

## 1. Introduction

The tomato leaf curl New Delhi virus (ToLCNDV) is a bipartite, single-stranded DNA begomovirus (genus *Begomovirus*, familiy *Geminiviridae*) that was first identified in India in 1995 affecting solanaceous crops [1] and thereafter causing major damage to cucurbit crops on the Indian subcontinent [2]. ToLCNDV was first detected in Europe in 2012, affecting zucchini squash, melon, and to a lesser degree tomato, in Spain [3,4]. Subsequently the virus was reported from Estonia, Greece, Italy, Portugal, Spain, Tunisia, Morocco, and Algeria [5]. The latest studies of ToLCNDV from Spanish isolates provide evidence that it is a new strain, denominated ToLCNDV-ES, that may have evolved by recombination [6,7]. Since the apparent speed by which ToLCNDV spreads to different countries and the extent of several outbreaks in countries such as Spain and Italy [8,9], the virus is listed as a quarantine pest by EPPO [5]. Symptoms that are produced by ToLCNDV-ES in zucchini are curling, chlorosis and vein thickening of leaves, stunted growth, and fruit deformation and abortion, whereas in tomato the symptoms in leaves are reminiscent of tomato yellow leaf curl disease [6]. ToLCNDV-ES genome titres that are detected are significantly lower in tomato than in zucchini plants, which may be related to the dissimilarities in symptom expression, capability of detection, and transmission of the virus [10]. Although ToLCNDV transmission through seeds and mechanical transmission have been experimentally shown to be possible, they are expected to be of minor significance under field conditions [11]. Instead, ToLCNDV-ES is reported to be transmitted by Mediterranean-Q1 *Bemisia tabaci* cryptic species in Spain, and probably by the Med-Q2 species in Italy [12,13].

The Mediterranean country of Spain has over 55,000 ha of tomato, 18,000 ha of muskmelon, and 11,000 ha of zucchini (data from 2020 from https://www.mapa.gob.es). Significant portions of these crop surfaces are very near to one another and are located within the same regions such as the south-east of Spain, where ToLCNDV-ES and its vector, *B. tabaci* cryptic species Q1 are highly adapted [12]. The presence of a potentially broad host range within the same geographic region jeopardizes economically important crops in the region [14] and could make the control of plant viruses difficult. *B. tabaci*-transmission of ToLCNDV-ES between tomato and zucchini has been reported [6], but details on the major parameters of the transmission and unknown. Viruses that belong to member species of the genus *Begomovirus* are considered as being transmitted in a non-propagative, persistent, circulative manner by the whitefly *B. tabaci*. Circulative, non-propagative viruses do not replicate in vector tissues, but traverse the insect gut, hemolymph, and salivary tissue membranes to reach the salivary glands for transmission [15]. Following acquisition, virions from many begomovirus species can be detected often in the whitefly vector for its entire life [16], yet the efficiency of transmission decreases with an increase in whitefly age and is negatively correlated with the amount of virus that is detectable in the vector.

Differences in the transmission of ToLCNDV-ES between different host species could yield epidemiological knowledge that could predict the success of control measures within multicrop horticulture regions. This also refers to the management of infected residues where plants that are removed from greenhouses after the crop harvest has finished that are often loaded with *B. tabaci* immature stages. Traditionally these crop remains are considered to be a source of infection, but the infectivity for ToLCNDV of emerging *B. tabaci* adults has not been proven. This particular aspect of virus retention in the whitefly is not only important for efficient management of the short-distance spread of the virus, but it also would be relevant to its long-distance spread. In fact, as part of the regulation of the virus under Commission Implementing Regulation (EU) 2019/2072, the main pathway that was identified for long-distance spread of the virus and of entry in new geographic regions are plants for the planting of susceptible hosts and consist of commodities carrying viruliferous *B. tabaci*.

Here we compare the parameters of efficiency, minimum acquisition access period (AAP), and inoculation access period (IAP) of ToLCNDV-ES transmission in zucchini and tomato, and we evaluated the retention in the vector. In addition, we determined the viral loads in tomato and zucchini, as well as in *B. tabaci*, feeding as adults, or emerging from pupae on infected tomato and zucchini, and we compared the infectivity of the emerging adults from both plant species.

## 2. Results

### 2.1. ToLCNDV Transmission in Zucchini and Tomato

The proportions of plants that were infected with ToLCNDV-ES following the introduction of increasing numbers of viruliferous adults *B. tabaci* whiteflies were compared. The transmission efficiencies in tomato and zucchini differed for increasing numbers of viruliferous *B. tabaci* and plant species. All of the 100 zucchini plants were infected after transferring 2, 5, and 20 *B. tabaci* per plant, as they all were symptomatic and had detectable ToLCNCV DNA as determined by conventional PCR. When they were tested with single viruliferous *B. tabaci*, 96% of zucchini plants showed the typical symptoms and were positive for ToLCNDV. In contrast, single *B. tabaci* whiteflies or groups of 2 of viruliferous whiteflies did not transmit the virus to any of 100 tomato plants. Feeding tomato plants with groups of 5, 20, and 50 viruliferous *B. tabaci* resulted in 15, 30, and 100% of symptomatic and virus positive tomato plants, respectively (Table 1).

The efficiency of transmission of *B. tabaci* adults that had fed on infected plants contrasted with that from adults that emerged from pupae that were reared on infected plants. Only 4 out of 20 *B. tabaci* adults that were collected immediately after emerging from the pupal stages on infected zucchini plant leaves were found to infect virus-free zucchini plantlets in transmission experiments using one adult insect per plant. In contrast, no transmission to zucchini was found by 20 individual adults that had emerged from pupae on infected tomato leaves at the same insect/plant rate.

### 2.2. Inoculation Access Period (IAP)

The minimum IAP was estimated using virus-free *B. tabaci* that was transferred for feeding during 72 h on ToLCNDV- infected zucchini plants. Next, groups of 20 viruliferous adult *B. tabaci* whiteflies were transferred onto virus-free zucchini and tomato plants. After periods ranging from 5 min to 24 h, they were removed, and 21 days later, apical leaf samples were removed and tested by conventional PCR. In three independent experiments, viruliferous *B. tabaci* infected 53% of zucchini plants and 43% of tomato plants following 5 min of feeding with viruliferous *B. tabaci*. Inoculation periods of 15 min were required to infect 90% of zucchini plants but the proportions of the infected tomato plants were about 50%. Higher proportions of tomato plants were infected after increasing inoculation feeding periods, but never exceeded 80% of the plants (Table 2.)

### 2.3. Acquisition Access Period (AAP)

The minimum AAP that was required for transmission was determined by allowing groups of 50 virus-free *B. tabaci* whiteflies in clip-cages to feed on ToLCNDV-infected zucchini and tomato plants. After periods of 5, 15, 30, and 60 min, and 6 h, they were transferred to virus-free zucchini plants. After another 48 h, they were removed and 19 days later and plants were analyzed by PCR. No transmission of ToLCNDV into zucchini was observed after five min of acquisition feeding. Limited numbers of zucchini plants got infected after 15 and 30 min of acquisition (20–60%) from the infected zucchini and tomato plants, and all the plants got infected after 6 h of acquisition feeding period from both source plant species (Table 3).

### 2.4. Persistence of Virus Infectivity in B. tabaci Adults

Virus-free *B. tabaci* adults were given a 72 h AAP on ToLCNDV-infected zucchini and then transferred to eggplant, and then as groups of 20 insects were transferred immediately to virus-free zucchini plants. Next, groups of 20 insects were transferred every two days from the eggplants onto virus-free zucchini. For three weeks after transmission, all the zucchini plants were observed for the expression of symptoms and tested for the virus by conventional PCR. It showed that viruliferous *B. tabaci* infected zucchini plants after periods of up to 14 days maintained on eggplant. Most of the insects retained infectivity for two days, which then gradually reduced and was completely lost after 9, 14, and 16 days, in three independent experiments (Table 4).

### 2.5. ToLCNDV Accumulation in Zucchini, Tomato, and B. tabaci

**Figure 1:**
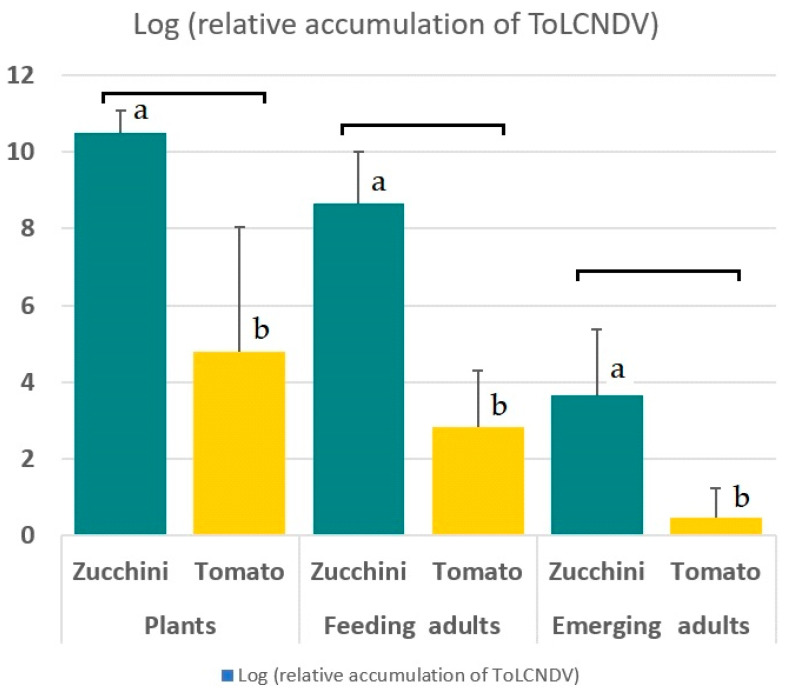
Log-transformed ToLCNDV accumulation in infected zucchini and tomato plants, in *B. tabaci* adults feeding on infected plants, and in adults emerging from pupae that were collected from dried leaves of infected zucchini and tomato plants. The mean values of 50 replicates in each group; the bars represent S.D. The letters represent the statistical significant difference (*p* < 0.05) using the Wilcoxon test.

The mean relative accumulation of ToLCNDV was 3.08 × 10^10^ in zucchini and 6.25 × 10^4^ in tomato plants. All plants that were infected with ToLCNDV had detectable amounts of virus. The viral loads of 10 infected zucchini plants ranged from 6.11 × 10^9^ to 4.39 × 10^11^, with a mean value of 7.86 × 10^10^. All 50 *B. tabaci* adults that were collected from these ToLCNDV-infected zucchini plants were positive for the virus with viral loads ranging from 7.64 × 10^4^ to 2.47 × 10^10^, with a mean value of 3.49 × 10^9^. A minimum viral load of 10^5^ was found in 98% of these whiteflies. When leaves containing the immature stages of the vector were removed and dried, emerging adults were collected and the ToLCNDV was determined. From 50 emerging adults, 40 (80%) of those that were tested were positive and 10 (20%) were negative. The positive *B. tabaci* adults had viral loads ranging from 1.82 × 10^2^ to 1.51 × 10^7^. Among the emerging vectors from zucchini leaves, 13 adults had a viral load that was higher than 10^5^, which is equivalent to 26% of all emerging individuals of infected zucchini (Figure 1).

All the tomato plants that were infected with ToLCNDV had detectable amounts of the virus, but the amounts were very variable and ranged from 7.54 × 10^1^ to 1.93 × 10^9^, with a mean value of 8.92 × 10^8^. In contrast to what was observed in the zucchini, approximately 10% of the 50 *B. tabaci* adults that were collected from the infected tomato tested negative for ToLCNDV by qPCR. The remaining 90% of the insects had detectable amounts of virus, ranging from 1.41 × 10^1^ to 2.31 × 10^6^, and 95% of these positive insects had viral loads below 10^5^. The Wilcoxon test for the comparison of independent populations comparing the accumulation values of ToLCNDV for adults that were collected from infected zucchini (median relative viral load 9.90 × 10^8^) and from tomato (median relative viral load 2.51 × 10^3^) was significantly different (*p* < 0.05, with a confidence level of 95.0%). From 50 emerging adults, 13 (26%) out of the 50 that emerged from the dried leaves of infected tomato tested negative. The positive *B. tabaci* had viral loads ranging from 1.02 to 4.68 × 10^2^ (Figure 1).

## 3. Discussion

The whitefly *B. tabaci* transmits more than 100 species of viruses to plants. The mode of the transmission is related to the taxonomical status of the viruses, and is characterized as semipersistent (criniviruses, ipomoviruses, and torradoviruses) and persistent (begomoviruses) [17]. These viruses generally differ in the values of the transmission parameters, such as: the probability of viruliferous insects to infect a plant, the time it takes a vector to ingest virus and to inoculated the virus into plants by feeding, and in the time the whiteflies remain infective following acquisition of the virus. In the present paper, we have compared the parameters of transmission of the bipartite begomovirus ToLCNDV-ES by the vector, *B. tabaci* Med-Q1, in two different crop species, tomato and zucchini.

Single viruliferous *B. tabaci* adults infected 96% of zucchini plants and none of 100 tested tomato plants. At least five adults were required to achieve infection in 15% of tomato (Table 1). The transmission efficiencies that were reported for several other bipartite begomovirus pathosystems in tomato are different, e.g., 45–60% for tomato leaf curl Gujarat virus [18], or 42% for tomato severe rugose virus (ToSRV), and 8% for tomato golden vein virus (TGVV) [19]. In contrast, when comparing the IAP and AAP of the transmission of ToLCNDV in zucchini and tomato, they were similar for both host-plant species: 5 and 20 min (Table 2 and Table 3), respectively. These values fall within the range of those that were reported for ToLCNDV in bottle gourd, which were estimated to be 10 and 30 min respectively [20]. They are also similar to other begomoviruses, such as tomato leaf curl virus from Bangalore (ToLCV-Ban4) in tomato where minimum AAP and IAP were 10 min and 20 min, respectively [21].

A single insect is able to acquire monopartite begomovirus TYLCV and transmit it to tomato plants. The reported minimum AAP and IAP of TYLCV isolates by *B. tabaci* biotype B (MEAM1) varies from 15 to 60 min and from 15 to 30 min, respectively [22,23,24]. Similar values were reported for other monopartite geminiviruses infecting tomato such as TYLCV Sardinia virus (TYLCSV) from Italy [25]. The mean infectivity of infected *B. tabaci* adults was retained during 7 to 14 days (Table 4). In general, begomoviruses are retained in the vector for nearly of almost its entire life. Adults of MEAM1 *B. tabaci* that acquired tomato severe rugose virus (ToSRV) during an AAP of 24 h on infected tomato remained viruliferous for 25 days, the maximum period that the insects survived when kept on cabbage plants that are immune to the virus [26]. Tomato leaf curl Sinaloa virus (TOLSCI) was detected in adults of *B. tabaci* up to nine days [27]. However, the squash leaf curl virus (SLCV) was retained in the insect for 26 days [28].

Thus, the maximum retention of 14 days has been measured after a six-hour acquisition period [29]. The retention values of 20 days have also been reported for Chino del tomato virus (CdTV) and tomato yellow vein streak virus (ToYVSV) [30,31], and life-long retention in the vector was reported for tomato yellow leaf curl Thailand virus (TYLCTHV) [32]. Comparisons of the transmission of ToLCNDV-ES and other begomoviruses should be done with caution. Despite differences in vector species, plant host species, and in the experimental circumstances, the values for IAP, AAP, and the persistence of ToLCNDV-ES were similar to those of monopartite and other bipartite begomoviruses. Transmission efficiencies, however, were different, and in other begomovirus pathosystems, this type of difference has been shown to be associated with feeding habits and preferences on the plant hosts that were used for acquisition and transmission [33,34,35,36]. The efficiency is further complicated by differences in the amount and distribution of begomoviruses in the different plant hosts being studied [37]. Also, the presence of selected endosymbionts (ex. *Hamiltonella* spp.) has been shown to affect the transmission efficiency of begomoviruses [38,39]. Finally, ToLCNDV-ES in Mediterranean countries occurs in cucurbitaceous crops that are potentially co-infected with *B. tabaci*-transmitted cucurbit yellow stunting disorder crinivirus (CYSDV) and cucumber vein yellowing ipomovirus (CVYV) [40]. These semipersistant transmitted viruses have no latent period after ingestion and the retention in the vector lasts from hours to days, depending on the species [41]. There is no evidence of an interference between CYSDV and CVYV in the transmission by *B. tabaci* [42,43], but the effect of coinfecting plant viruses on vector-transmission has been suggested in other combinations of mixed infections, i.e., of CYSDV and aphid-transmitted watermelon mosaic virus (WMV) [44]. Therefore, the effect of criniviruses and ipomoviruses on the transmission of ToLCNDV-ES requires further investigation.

The efficiency of ToLCNDV-ES transmission by single insects was low in tomato and very high in zucchini. These differences in the inoculation efficiency were used to explain the predominance of different begomoviruses that are acquired at similar rates by the same vector species in the same crop species, such as tomato severe rugose virus (ToSRV) versus tomato golden vein virus (ToSRV) in Brazilian tomato fields [19,45]. But transmission efficiencies may also reflect host plant resistance and the ability of a virus to replicate inside the host plant [46]. The higher the host plants resistance, the lower the transmission rate and the lower is the amount of virus that is detected in the host plant. This has been shown for TYLCV where viral DNA accumulation was shown to be lower in the resistant source plants compared with the susceptible plants [47].

ToLCNDV-ES has been found as natural and experimental infections in cucurbit and solanaceous species [4,8]. However, relative incidences of the virus are very different among crops in the same agronomic region of the south-east of Spain. Among commercial crops, the percentages of plants that are infected with ToLCNDV varied from 95% in zucchini, 80% in melon, 50% in cucumber, 0% in watermelon, and 15% in tomato [8]. Viral loads in tomato that were experimentally infected with ToLCNDV-ES were also reduced when compared with zucchini (Figure 1). This conforms to previously published comparisons of the viral loads of ToLCNDV-ES in zucchini and in tomato [10]. As such, both the natural incidences of ToLCNDV-ES in zucchini and tomato, the viral loads in this paper, and those that were published before suggest that these are in line with the significantly different efficiencies of transmission in both crop species. Since the transmission efficiency in zucchini is almost 100% for single insects, and because the disease can be fatal in this crop species, control of ToLCNDV in zucchini is a big challenge, and requires efficient physical vector exclusion using greenhouses and careful planning and application of biological and integrated pest management [48,49]. Moreover, the success of these control strategies could change whenever evidence of differences in the ToLCNDV-ES transmission by co-infecting criniviruses or ipomoviruses is obtained, following the future research as suggested above.

Natural incidences of ToLCNDV-ES are lower in commercial tomato crops [8], which is consistent with the reduced transmission efficiency in this host plant (Table 1). However, crop protection against *B. tabaci* and ToLCNDV should also be applied with care in tomato because these plants may often be co-infected with other begomoviruses, such as monopartite TYLCV species [10], which may compromise the interpretation of the observed symptoms.

The present paper established the retention of ToLCNDV in adult vectors to be around between 7 and 14 days, but here we also determined a different aspect of virus retention: we showed that 20% of adults that emerged from pupae on drying zucchini plant leaves were actually infective. This ratio is comparable to TYLCV in tomato where 28% of emerging adults were found to be infective [30]. We also established that the amounts of the virus in emerging adults (ranging between approx. 10^2^ and 10^7^), were generally lower that the amounts in the plants (between approx. 10^9^ and 10^11^). Since the 20% higher virus levels in adults from the dried zucchini leaves were approx. 10^5^ or more, this value may well represent the minimum viral load in vectors that is necessary to achieve a successful infection. In comparison, all single adults that were collected from infected zucchini plants had values above 7 × 10^4^, and 96% of single adults that successfully infected zucchini. In contrast, none of adults emerging from pupae on the infected dried tomato leaves were able to infect zucchini plants, and 95% of these *B. tabaci* had viral loads below 10^5^.

The efficiency of transmission and the viral loads in adults that were emerging from pupae may explain why biological control using predators of *B. tabaci* eggs and immature stages can significantly reduce the short-distance spread of the virus in greenhouses [48,49]. However, it may be also significant in the long-distance control of ToLCNDV-ES, i.e., in the trade and transport of infected plant material, when no *B. tabaci* adults are spotted, these commodities may contain small-sized immature stages that could well be a source of infection [50]. On the other hand, the infectivity of emerging adults from infected dried plant materials provides evidence that plants that are removed from the crop either during the growing and harvesting stage, or after the crops have finished, should be carefully covered, in sealed boxes, containers, etc., and carefully treated or destroyed, but not exposed or transported as such as that would permit the spread of emerging viruliferous vectors and, consequently, the spread of the virus. So, ToLCNDV-ES management of crops not only should involve hygiene, vector exclusion, and control during the nursery, production, and harvesting stages, but also once the production and harvesting have finished and the plants are removed from the field or the greenhouse, because even as dried materials, they can contain immature vector stages and produce viruliferous emerging insects.

## 4. Materials and Methods

### 4.1. Host Plants, B. tabaci Populations and Virus Isolate

Experiments were carried out using zucchini (*Cucurbita pepo* cv. ‘Victoria’) and tomato (*Solanum lycopersicum* “Marmande”). A virus-free population of *B. tabaci* that were originally collected from a commercial eggplant crop (*Solanum melongena*) in spring of 2012, was reared on eggplant (cv. Crisol) in insect-proof cages in a growth chamber at 25 °C day and 20 °C night, with a 16-h photoperiod. This population was identified as the Mediterranean cryptic species subclade Q1 [12]. For the transmission experiments, *B. tabaci* adults of the same age were obtained by collecting the insects that emerged during a given 24-h period. ToLCNDV-ES that was originally isolated during 2013 from zucchini that were located in a commercial greenhouse from Almeria, Spain, was maintained on zucchini (*C. pepo* cv. ‘Victoria’) with *B. tabaci*, grown in insect cages as described [6]. The plants and *B. tabaci* whiteflies were analysed by PCR to ensure the presence of ToLCNDV-ES (primers and protocols are described below), and the absence of cucurbit viruses that are transmitted by *B. tabaci* in Spain, cucurbit yellow stunting disorder virus and cucumber vein yellowing virus, was confirmed by RT-PCR as described [42].

### 4.2. Virus Detection and Quantification

DNA extracts from zucchini and tomato leaf samples were obtained with the DNeasy Plant kit (QIAGEN, Madrid, Spain), whereas the nucleic acid extracts of *B. tabaci* adults were prepared using Chelex 100 [51]. Conventional PCR for ToLCNDV was carried out using the primer pairs A1UP/A3LOW (5′-AGCACAGCCACGGTGAAGAAC-3′/5′-TTTCATCCTTCGACAGAGTTC-3′) and B4UP/B4LOW (5′-ATGTAATTGGTGTCTGGAGTCC-3′/5′-TTAACCTGATGTAGGAACGAACG-3′) that were designed based on ToLCNDV sequences that are deposited in GenBank KF89146 and KF891467, respectively. These primer pairs amplify parts of the DNA-A and DNA-B components (1260 and 873 bp, respectively) [6]. The conventional PCR reagent was Applied Biosystems AmpliTaq DNA Polymerase (ThermoFisher Scientific, Madrid, Spain) which was used as described with an annealing temperature of 55 °C [6], and determinations were done in an Eppendorf MasterCycler Personal PCR Thermal Cycler (Eppendorf Iberica S.L.U., Madrid, Spain). The viral load in plant extracts was determined by qPCR using TolAup (5′-CATTATTGCACGAATTTCCG-3′)/TolAdown (5′-ATCGTAGCCGACTGTGTCTG-3′) primers as described before [10] but using actine specific primers ActineF (5′-GATGGACAAGTCATCACCATTG-3′) and ActineL (5′-CTGAGGACAATGTTTCCGTACA-3′) that produced 151 bp product from the actin gene (AY594294) for internal control [52]. For quantification of ToLCNDV in *B. tabaci* DNA extracts, identical primers were used for the detection of the virus, and primers Bemisia-3F (5’-AAGGATCATTGTCGAACTCGA-3’) and 65R (5’-CCTGTGTCCCGCGGG-3’) were used as internal controls [43]. The qPCR reagent was qMAXSen™ Green Dye qPCR Master Mix2x (CANVAX, Cordoba, Spain), and determinations were done in a LightCycler 96 (Roche, Madrid, Spain). The relative ToLCNDV levels were calculated using the 2−ΔΔCt expression of the Livak method [53], where ΔΔCt is the difference between the ΔCt of each sample and the ΔCt of the calibrator sample.

### 4.3. Virus Transmission Parameters Using B. tabaci

**Figure 2:**
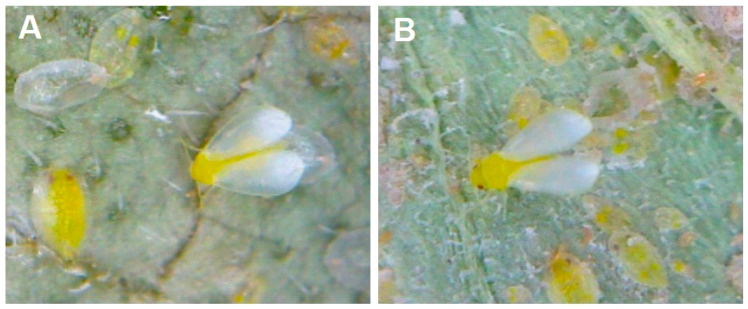
Adult of *B. tabaci* that were recently emerged from pupae that were developed on ToLCNDV-ES-infected zucchini (**A**) and tomato (**B**) leaves.

For the estimation of the transmission efficiency, newly emerged (0–24 h) *B. tabaci* adults were allowed to feed on ToLCNDV-ES-infected zucchini plants as single insects or in groups of 2, 5, 20, and 50 insects (selected at random) for a 72-h acquisition access period (AAP) and then transferred onto virus-free zucchini and tomato target plants (three-leaf stage), that were grown in individual insect-proof cages, for virus transmission. A total of three weeks later, the plants were observed for symptoms and tested by conventional PCR. The persistence of the infectivity of adults that were emerging from pupae that were reared on infected plants was determined as follows: the plant leaves with immature stages of *B. tabaci* were collected from ToLCNDV-infected zucchini and tomato plants and kept at 25 ºC in plastic cages, covered with an insect screen. As the leaves dried out, they were continuously inspected and 20 adults that were emerging from the pupae stage from both plant species each, were collected (Figure 2, Videos S1 and S2) and were transferred as single insects in clip-cages onto virus-free zucchini plants (1 clip-cage/plant). After three weeks, the plants were analysed.

The minimum inoculation access period (IAP) was estimated using virus-free *B. tabaci* that were transferred for feeding during 72 h on ToLCNDV-infected zucchini plants. Next, groups of 20 viruliferous *B. tabaci* adults were transferred onto virus-free zucchini and tomato plants. After periods ranging from 5 min to 24 h, they were removed, and 21 days later, the apical leaf samples were removed and tested by conventional PCR. A total of three independent experiments were performed.

The minimum AAP that was required for transmission was determined by allowing groups of 50 virus-free *B. tabaci* in clip-cages to feed on ToLCNDV-infected zucchini and tomato plants. After periods of 5, 15, 30, and 60 min, and 6 h, they were transferred to virus-free zucchini plants. After another 48 h, they were removed and 19 days later the plants were analyzed by conventional PCR.

To evaluate the persistence of the infectivity of the vector following acquisition by feeding as adult whiteflies, virus-free *B. tabaci* adults were given a 72 h AAP on ToLCNDV-infected zucchini and then transferred to eggplant. Groups of 20 insects were transferred immediately to virus-free zucchini plants, and then groups of 20 insects were transferred every two days from the eggplants onto virus-free zucchini. For three weeks after transmission, all zucchini plants were observed for the expression of symptoms and tested for the virus by PCR.

### 4.4. ToLCNDV Accumulation in Zucchini, Tomato, and B. tabaci

To estimate and compare the accumulation of ToLCNDV in the two hosts plant species as well as in the vector, two groups of 50 virus-free *B. tabaci* adults were allowed to feed during 3 weeks on 10 ToLCNDV-infected zucchini and tomato plants in separate insect-cages. The insects were removed, and the DNA was extracted from each adult individual, as well as from each tomato and zucchini plant as described above. The qPCR reactions were done as described above. In addition, the viral loads were also determined in adults that were recently emerged from infected plants that were collected as described above. Here, the adults emerging from the pupae stage were collected using a Pasteur pipette and stored at -20 ºC prior to DNA extraction and qPCR.

## Figures and Tables

**Table 1:** Transmission efficiency of ToLCNDV-ES to zucchini and tomato by *B. tabaci* ^a^.

Plant Species	Number of Viruliferous *B. tabaci*				
	1	2	5	20	50
Zucchini	96 (96) ^b^	100 (100)	100 (100)	100 (100)	Not done
Tomato	0 (0)	0 (0)	15 (15)	30 (34)	100 (100)

^a^ *B. tabaci* whiteflies were allowed a 72 h virus AAP, and then transferred onto virus-free seedlings for 96 h. Each experiment was conducted on groups of 100 plants. ^b^ percentages of plants expressing ToLCNDV symptoms (percentages of positive plants tested with conventional PCR).

**Table 2:** Inoculation feeding period of the transmission of ToLCNDV by *B. tabaci.*

IAP	Host Plant Species								
	Tomato				Zucchini				
	Exp. 1	Exp. 2	Exp. 3	Mean ± S.D.	Exp. 1	Exp. 2	Exp. 3	Mean ± S.D	*p*-Value ^b^
0 min	0 ^a^	0	0	0.00 ± 0.00	0	0	0	0.00 ± 0.00	-
5 min	4	4	5	4.33 ± 0.58	5	6	5	5.33 ± 0.58	0. 101
15 min	5	4	6	5.00 ± 1.00	9	10	9	9.33 ± 0.58	0.003
30 min	3	6	5	4.67 ± 1.53	10	9	10	9.67 ± 0.58	0.006
60 min	7	7	7	7.00 ± 0.00	10	9	10	9.67 ± 0.58	0.001
6 h	4	5	7	5.33 ± 1.53	8	10	9	9.00 ± 1.00	0.025
16 h	8	6	8	7.33 ± 1.15	9	10	10	9.67 ± 0.58	0.035
24 h	6	8	7	7.00 ± 1.00	10	10	9	9.67 ± 0.58	0.016

^a^ Numbers of plants (total = 10) positive por the virus by PCR, following different feeding periods; ^b^ A two-tailed *t*-test was applied for comparisons of differences between the numbers of infected tomato and zucchini plants. *p*-value < 0.05 considered as statistically significant; S. D., standard deviation.

**Table 3:** Acquisition access period of the transmission of ToLCNDV-ES by *B. tabaci*.

Source Species	AAP (min)				
	5	10	30	60	360
Zucchini	0 ^a^	20	60	80	100
Tomato	0	20	20	40	100

^a^ Percentages in groups of 50 target plants (zucchini) positive for ToLCNDV-ES.

**Table 4:** Persistence of infectivity by adults *B. tabaci* whiteflies.

Days	Number of Plants Infected with ToLCNDV-ES			Mean ± S.D.	Mean % ± S.D
	Experiment 1	Experiment 2	Experiment 3		
0 ^a^	20 ^b^	18	20	19.33 ± 1.15	96.67 ± 5.77
2	20	18	20	19.33 ± 1.15	96.67 ± 5.77
5	20	10	16	15.33 ± 5.03	76.67 ± 25.17
7	18	8	16	14.00 ± 5.29	70.00 ± 26.46
9	4	0	6	3.33 ± 3.06	16.67 ± 15.28
12	4	0	2	2.00 ± 2.00	10.00 ± 10.00
14	4	0	0	1.33 ± 2.31	6.67 ± 11.55
16	0	0	0	0.00 ± 0.00	0.00 ± 0.00
19	0	0	0	0.00 ± 0.00	0.00 ± 0.00
21	0	0	0	0.00 ± 0.00	0.00 ± 0.00

^a^ Days of *B. tabaci* transfer after 48 h AAP; ^b^ numbers of zucchini plants infected out of 20.

## Data Availability

Data is contained within the article.

## Supplementary Materials

The following are available online at https://www.mdpi.com/article/10.3390/plants11030390/s1, Video S1: *B. tabaci* adult whitefly emerging from pupae on a ToLCNDV-infected zucchini leaf, Video S2: *B. tabaci* adult whitefly emerging from pupae on a ToLCNDV-infected tomato leaf.
